# Structural Brain Damage and Upper Limb Kinematics in Children with Unilateral Cerebral Palsy

**DOI:** 10.3389/fnhum.2017.00607

**Published:** 2017-12-12

**Authors:** Lisa Mailleux, Cristina Simon-Martinez, Katrijn Klingels, Ellen Jaspers, Kaat Desloovere, Philippe Demaerel, Simona Fiori, Andrea Guzzetta, Els Ortibus, Hilde Feys

**Affiliations:** ^1^Department of Rehabilitation Sciences, KU Leuven, Leuven, Belgium; ^2^BIOMED, Rehabilitation Research Center (REVAL), Hasselt University, Diepenbeek, Belgium; ^3^Neural Control of Movement Lab, Department of Health Sciences and Technology, ETH Zurich, Zurich, Switzerland; ^4^Clinical Motion Analysis Laboratory, University Hospitals Leuven, Leuven, Belgium; ^5^Department of Imaging and Pathology, KU Leuven, Leuven, Belgium; ^6^IRCCS Stella Maris Foundation, Pisa, Italy; ^7^Department of Clinical and Experimental Medicine, University of Pisa, Pisa, Italy; ^8^Department of Development and Regeneration, KU Leuven, Leuven, Belgium

**Keywords:** upper extremity, cerebral palsy, magnetic resonance imaging, brain injuries, biomechanical phenomena

## Abstract

**Background:** In children with unilateral cerebral palsy (uCP) virtually nothing is known on the relation between structural brain damage and upper limb (UL) kinematics quantified with three-dimensional movement analysis (3DMA). This explorative study aimed to (1) investigate differences in UL kinematics between children with different lesion timings, i.e., periventricular white matter (PWM) vs. cortical and deep gray matter (CDGM) lesions and (2) to explore the relation between UL kinematics and lesion location and extent within each lesion timing group.

**Methods:** Forty-eight children (age 10.4 ± 2.7 year; 29 boys; 21 right-sided; 33 PWM; 15 CDGM) underwent an UL 3DMA during a reach-to-grasp task. Spatiotemporal parameters [movement duration, (timing of) maximum velocity, trajectory straightness], the Arm Profile Score (APS) and Arm Variable Scores (AVS) were extracted. The APS and AVS refer to the total amount of movement pathology and movement deviations of the wrist, elbow, shoulder, scapula and trunk respectively. Brain lesion location and extent were scored based on FLAIR-images using a semi-quantitative MRI-scale.

**Results:** Children with CDGM lesions showed more aberrant spatiotemporal parameters (*p* < 0.03) and more movement pathology (APS, *p* = 0.003) compared to the PWM group, mostly characterized by increased wrist flexion (*p* = 0.01). In the CDGM group, moderate to high correlations were found between lesion location and extent and duration, timing of maximum velocity and trajectory straightness (*r* = 0.53–0.90). Lesion location and extent were further moderately correlated with distal UL movement pathology (wrist flexion/extension, elbow pronation/supination, elbow flexion/extension; *r* = 0.50–0.65) and with the APS (*r* = 0.51–0.63). In the PWM group, only a few and low correlations were observed, mostly between damage to the PLIC and higher AVS of elbow flexion/extension, shoulder elevation and trunk rotation (*r* = 0.35–0.42). Regression analysis revealed damage to the temporal lobe with lesion timing as interactor (27%, *p* = 0.002) and the posterior limb of the internal capsule (PLIC) (7%, *p* = 0.04) as the strongest predictors, explaining 34% of the variance in APS.

**Conclusion:** UL kinematic deviations are more influenced by lesion location and extent in children with later (CDGM) versus earlier lesions (PWM), except for proximal movement pathology. Damage to the PLIC is a significant predictor for UL movement pathology irrespective of lesion timing.

## Introduction

The ability to efficiently coordinate trunk, arm and hand movements is crucial in order to successfully reach and grasp and execute daily life activities such as eating, personal hygiene and self-care. In children with unilateral cerebral palsy (uCP), the presence of pathological movement patterns at the impaired upper limb (UL) has been shown to be associated with lower levels of unimanual capacity and bimanual performance impeding activities of daily life (Mailleux et al., 2017a). In these children, UL movement pathology is a common feature during the execution of various tasks, characterized by increased wrist and elbow flexion and elbow pronation accompanied by compensatory movements of the shoulder, scapula and trunk as assessed with three-dimensional movement analysis (3DMA) (Fitoussi et al., 2006; Jaspers et al., 2009, 2011a,c; Butler et al., 2010; Brochard et al., 2012; Butler and Rose, 2012; Klotz et al., 2014; Simon-Martinez et al., 2017). Still, virtually nothing is known about the underlying neuropathophysiology explaining UL movement pathology in children with uCP, while the relation between brain lesion characteristics and clinical outcomes of UL function has already been investigated (Feys et al., 2010; Holmström et al., 2010; Holmefur et al., 2013; Mackey et al., 2014; Mailleux et al., 2017b).

Brain lesions in children with uCP are often classified according to their presumed lesion timing: cortical maldevelopments (first and second trimester), periventricular white matter (PWM) lesions (early third trimester) and cortical and deep gray matter (CDGM) lesions (around term age) (Krägeloh-Mann and Horber, 2007). It has been shown that children with earlier lesions (i.e., PWM lesions) have a better UL function compared to children with lesions occurring later in life (i.e., CDGM lesions) (Feys et al., 2010; Holmström et al., 2010; Holmefur et al., 2013; Mackey et al., 2014). Apart from lesion timing, also lesion location and extent have been shown to relate with UL function, i.e., basal ganglia and/or thalamus involvement and more extended lesions are associated with a more impaired UL function (Feys et al., 2010; Holmström et al., 2010; Holmefur et al., 2013; Mackey et al., 2014; Shiran et al., 2014; Fiori et al., 2015; Baranello et al., 2016). We also recently demonstrated that lesion location and extent were more strongly related to UL function in children with CDGM lesions compared to children with PWM lesions (Mailleux et al., 2017b). Nevertheless, previous findings are based mainly on clinical outcomes, which do not provide information on selective anatomical motions at the individual joint levels. In contrast, an UL 3DMA can define movement deviations at joint level and provides an objective description of UL movement pathology.

So far, only Van Der Heide et al. (2005) investigated the relation between brain lesion severity and UL kinematics during a reaching task in children with both unilateral and bilateral CP. These authors demonstrated that more severe brain lesions were correlated with less straight hand trajectories. Nevertheless, in this study, kinematic outcomes were limited and brain lesions were visualized using ultrasound imaging, which has a lower spatial resolution and accuracy compared to MRI. In adult stroke patients, MRI research has shown that more severe cerebellar lesions (Konczak et al., 2010) and increasing brain activation in the ipsilesional motor cortex (Buma et al., 2016) correlate with poorer spatiotemporal parameters during reaching tasks. Interestingly, brain activation in the ipsilesional motor cortex was more strongly correlated with UL kinematics compared to clinical outcome measures of UL function (Buma et al., 2016). In addition, Meyns et al. (2016) also demonstrated the adverse impact of the underlying brain lesion on gait kinematics in children with CP. Together these findings point toward the benefits of UL 3DMA to further enhance our understanding of the complex interplay of structural brain damage and UL movement pathology in children with uCP.

Hence, this explorative study first aims to investigate whether UL kinematics differ between children with early (PWM) versus later lesions (CDGM) during a reach-to-grasp task. Secondly, we aim to explore the relation between lesion location and extent and UL kinematics within each lesion timing group. We hypothesize that children with CDGM lesions have more deviant UL kinematics than children with PWM lesions and that lesion location and extent impact more on UL movement pathology in children with CDGM compared to PWM lesions.

## Materials and methods

### Participants

Children were recruited via the CP-care program of the University Hospitals Leuven (Belgium). Children with a spastic type of uCP were enrolled in the study if they were aged between 5 and 15 years, able to comprehend test instructions, could at least actively grasp an object and had a brain MRI scan available taken after the age of 3 years. Additionally, only children classified with either PWM or CDGM lesions as defined by Krägeloh-Mann and Horber (2007) were included. Exclusion criteria were botulinum toxin-A injections 6 months prior to testing or a history of UL surgery. This study was carried out in accordance with the recommendations of the Medical Ethical Committee of the University Hospitals Leuven (S50480, S55555, and S56513). Written informed consent was obtained from all parents in accordance with the Declaration of Helsinki. In addition, children aged older than 12 years were asked for their assent prior to participation.

### Procedure

All children underwent an UL 3DMA at the Clinical Motion Analysis Laboratory of the University Hospitals Leuven. Children were assessed by well-trained physiotherapists routinely involved in the clinical evaluation of children with CP. Brain lesions were scored using a semi-quantitative MRI-scale (sqMRI scale, Fiori et al., 2014) by one pediatric neurologist (EO) who was blinded to the outcome of the UL 3DMA.

### Three-dimensional movement analysis

UL 3DMA was performed according to the protocol described by Jaspers et al. (2011b). Seventeen reflective markers were attached on the hand (*n* = 3), forearm (*n* = 4), humerus (*n* = 4), acromion (*n* = 3), and trunk (*n* = 3). The starting position was upright sitting with hips and knees in 90° flexion, ensured through a custom-made chair with adjustable foot and back support. Twelve to fifteen infrared Vicon-cameras were used for recordings, sampling at 100 Hz. First, static calibration trials were collected to identify the anatomical landmarks as described in Wu et al. (2005). Children were then asked to reach and grasp a vertically oriented cylinder (RGV). This cylinder was placed at shoulder height and arm length distance. The task RGV was chosen as it simultaneously requires elbow extension and supination, which adequately challenges the movement pattern of the UL in children with uCP. This task was executed twice with the impaired UL at self-selected speed. Each trial contained four movement repetitions, resulting in a total of eight movement repetitions. After data collection, two movement repetitions per trial were selected, depending on the child's task compliance and marker visibility (i.e., movement repetitions with marker occlusions >20% of the movement duration were excluded). Subsequently, start (i.e., hand on ipsilateral knee) and end positions (i.e., point of task achievement) of each movement repetition were identified using Nexus software (Oxford Metrics, Oxford, UK). Finally, UL kinematics were calculated in MATLAB using U.L.E.M.A. (v1.1.9, available for download^1^).

Spatiotemporal parameters and summary indices were extracted. Spatiotemporal parameters comprised movement duration, timing of maximum velocity, maximum velocity and trajectory straightness (i.e., calculated as the ratio of the actual length of the traveled hand path and the direct linear distance between start and endpoint). Summary indices included the Arm Profile Score (APS) and 13 Arm Variable Scores (AVS) and were determined as described in Jaspers et al. (2011c). The AVS was calculated for 13 joint angles as the root mean square error between the point-by-point comparison of each joint angle of the child with uCP and that same joint angle of a reference database (60 typically developing children, age 5–15 years). The root mean square error-average of all 13 joint angles equals the APS and represents the overall severity of UL movement pathology. The 13 AVS represent the deviating scores for the wrist (flexion/extension, ulnar/radial deviation), elbow (flexion/extension, pronation/supination), shoulder (elevation plane, elevation, rotation), scapula (anterior/posterior tilting, medial/lateral rotation, protraction/retraction) and trunk (flexion/extension, lateral bending, axial rotation).

### Semi-quantitative MRI scale

The available MRI scan included at least one FLAIR sequence and was taken after the age of 3 years as described by Fiori et al. (2014). First, the lesion was drawn onto a graphical template, adapted from the CH2 atlas (Mazziotta et al., 2001) using a red pen. This template consisted of six axial slices containing the drawing of three lines: a cortical outline, a subcortical line dividing the gray from the white matter and a periventricular line bordering the periventricular white matter resulting in a cortico-subcortical, middle white matter and periventricular white matter layer. The boundaries of the frontal, parietal, temporal and occipital lobes were marked according to the Talairach atlas (Talairach and Tournoux, 1988). Secondly, for both hemispheres each layer in each lobe was scored, resulting in a lobar score (range 0–3) and summed up to obtain a hemispheric score (range 0–12). Next, damage to five subcortical structures, i.e., lenticular and caudate nucleus, thalamus, posterior limb of internal capsule (PLIC) and brainstem, was scored directly from the MRI scan as affected (score 1) or not affected (score 0) (subcortical score, range 0–5). Damage to the corpus callosum (anterior, middle and posterior section, range 0–3) and cerebellum (vermis, right and left hemisphere, range 0–3) were also scored directly from the MRI scan. Subsequently, a contralesional and ipsilesional total score (range 0–17) were obtained as the sum of the hemispheric and subcortical score of each respective hemisphere. Finally, the sum of all these scores led to the global score (range 0–40). Reliability and validity of the scale has already been established in children with CP (Fiori et al., 2014, 2015).

Lesion location was defined as damage to each of the four lobes, three layers, and five subcortical structures of the ipsilesional hemisphere. Lesion extent was determined by the ipsilesional hemispheric score, ipsilesional subcortical score, ipsilesional total score and the global score.

### Statistical analysis

Descriptive statistics were used to document demographic and kinematic characteristics. First, all kinematic parameters were tested for normality using the Shapiro-Wilk test. Differences in UL kinematics between the PWM and CDGM group were investigated using unpaired *t*-tests or Mann-Whitney *U*-tests depending on the type of data. Correlation coefficients were calculated for both lesion timing groups separately, between kinematic parameters and the scores of ipsilesional brain damage, the contralesional total score, the corpus callosum and the global score using spearman (r_s_) or biserial (r_b_) correlation coefficients depending on the type of data. The cerebellum was excluded for further analysis, since none of the participants showed damage to this region. Due to the explorative nature of the study, no correction for multiple testing was applied (Bender and Lange, 2001). Hence, correlations will be discussed according to their strength. Correlation coefficients < 0.30 were considered as little or no correlation, 0.30–0.50 low, 0.50–0.70 moderate, 0.70–0.90 high, and >0.90 very high (Hinkle et al., 1998). Finally, a multiple regression analysis was used to identify the explained variance in UL movement pathology (APS). The variables entered into this model were selected via the Least Absolute Shrinkage and Selection Operator (LASSO) approach (Tibshirani, 1996). Variables entered in the LASSO approach were lesion timing, all ipsilesional scores for lesion location and extent, and age as well all ipsilesional scores with lesion timing as interacting variable. Two-sided 5% level of significance was used. Statistical procedures were carried out with SAS 9.4 (SAS Institute Inc., Cary, NC, USA).

## Results

### Participants

Forty-eight children (29 males, 19 females; 21 right-sided, 27 left-sided; Manual Ability Classification System, Eliasson et al., 2006; I = 11, II = 22, III = 15) with a spastic type of uCP were included in this study. Thirty-three children had PWM lesions and 15 had CDGM lesions. UL clinical characteristics according to lesion timing are presented in Supplementary Material ST1. Average age at time of the UL 3DMA was 10 years and 4 months (SD ± 2 years and 7 months). Age did not differ significantly between the PWM and CDGM group (*p* = 0.66).

### Differences in upper limb kinematics between lesion timing groups

**Figure 1:**
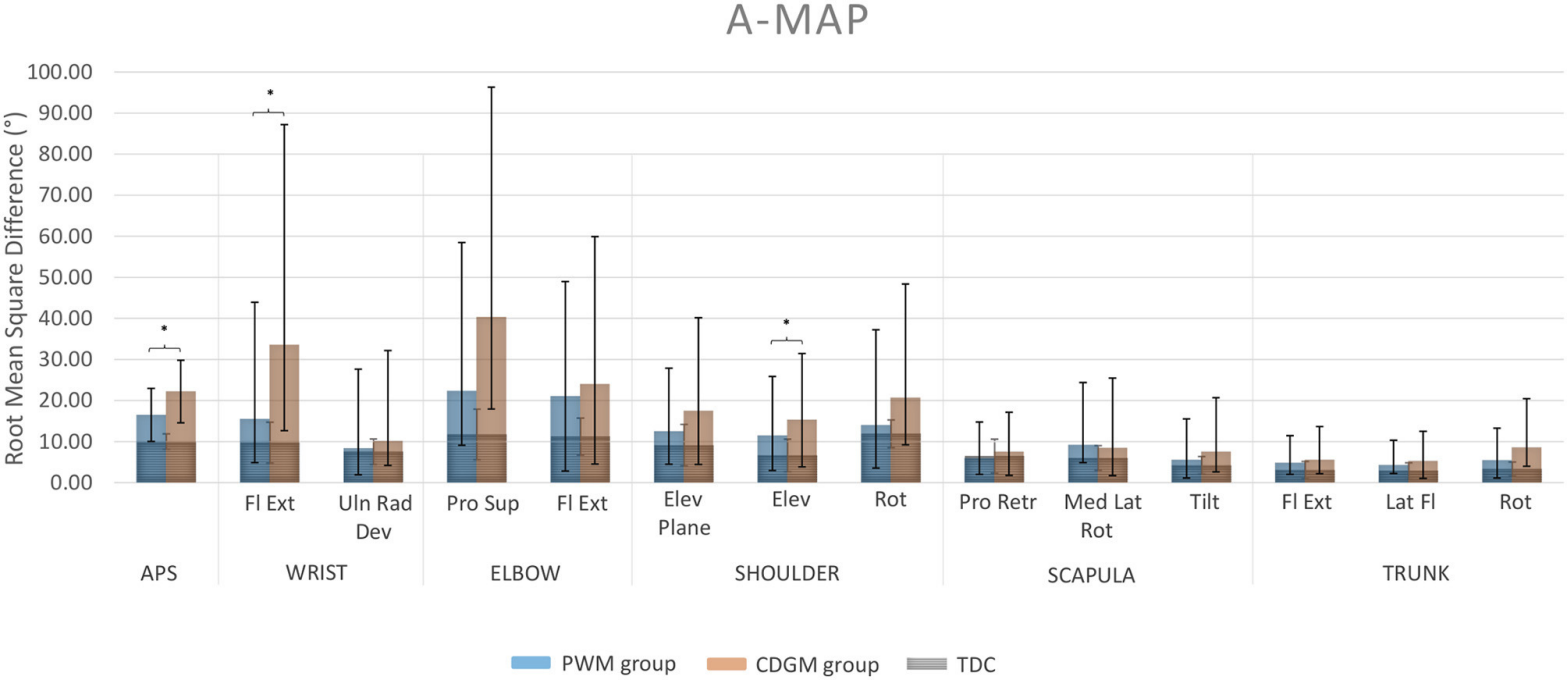
Arm Movement Analysis Profile (A-MAP) of the PWM and CDGM group. Each column represents the AVS of one specific joint angle. The APS for total upper limb movement pathology is presented in the leftmost column. Blue columns represent the AVS and APS of the PWM group; orange columns of the CDGM group. The AVS and APS of the typically developing group overlay the columns of both the PWM and CDGM group in gray. APS, Arm Profile Score; AVS, Arm Variable Score; Fl Ext, flexion/extension; Uln Rad Dev, ulnar/radial deviation; Pro Sup, pronation/supination; Elev, elevation; Rot, rotation; Pro Retr, protraction/retraction; Med Lat Rot, medial/lateral rotation; Tilt, tilting; Lat Fl, lateral flexion; PWM, periventricular white matter; CDGM, cortical and deep gray matter; TDC, typically developing children; ^*^significant difference between the PWM and CDGM group (*p* < 0.05).

Children with CDGM lesions had significantly longer movement durations (*p* = 0.03), earlier timings of maximum velocity (*p* = 0.0005) and less straight hand trajectories (*p* = 0.005) compared to children with PWM lesions (see Table 1). In addition, the scores for total movement pathology were higher in the CDGM group compared to the PWM group (APS, *p* = 0.003). Statistical comparison of the AVS further showed increased movement deviations of wrist flexion/extension (*p* = 0.01) and shoulder elevation (*p* = 0.05) and a trend for more deviating movement patterns of elbow pronation/supination (*p* = 0.08) in the CDGM group. The APS and 13 AVS for the PMW and CDGM group are presented in a bar chart, the Arm Movement Analysis Profile, which exhibits the contribution of each variable to the APS (A-MAP, Figure 1). Mean (standard deviation) and medians (interquartile ranges) per group can be found in Supplementary Material ST2.

**Table 1:** Statistical comparison of the spatiotemporal parameters in the PWM (*N* = 33) compared to the CDGM (*N* = 15) group.

		**PWM**	**CDGM**	***p*-value**
Duration (s)^a^	X (SD)	**1.56 (0.43)**	**1.94 (0.56)**	**0.03**
TimeVmax (%)^a^	X (SD)	**26.74 (5.09)**	**21.32 (4.25)**	**0.0005**
Vmax (m/s)^a^	X (SD)	1.05 (0.22)	1.15 (0.20)	0.12
TS^a^	X (SD)	**1.33 (0.23)**	**1.54 (0.23)**	**0.005**

a *unpaired t-test; X, mean; SD, standard deviation; TimeVmax, timing to maximum velocity; Vmax, maximum velocity; TS, trajectory straightness; PWM, periventricular white matter; CDGM, cortical and deep gray matter; bold indicates p < 0.05*.

### Relation between lesion location and extent and upper limb kinematics for each lesion timing group

#### Spatiotemporal parameters

In the **PWM group**, no correlations were found for the spatiotemporal parameters, except for two low correlations. Damage to the middle white matter layer and the PLIC were associated with longer movement durations (r_b_ = 0.31) and less straight hand trajectories (r_b_ = 0.33), respectively. Interestingly, in the **CDGM group** several moderate to high correlations were found between lesion location and extent and movement duration, timing of maximum velocity and trajectory straightness (r_b_ = 0.52 to −0.79), indicating longer movement durations, earlier timings of maximum velocity and less straight hand trajectories with increasing brain damage (see Table 2). In addition, one high correlation was found between higher contralesional total scores and earlier timings of maximum velocity (r_b_ = −0.71.) Regarding maximum velocity, only one moderate correlation was found, i.e., with damage to the corpus callosum (r_b_ = 0.62).

**Table 2:** Correlation coefficients between the spatiotemporal parameters and brain lesion scores in the CDGM group (*N* = 15).

	**Duration (s)^a^**	**TimeVmax (%)^a^**	**Vmax (m/s)^a^**	**TS^a^**
**LESION LOCATION**				
**Lobes**				
*Frontal (0–3)*	0.65^**^	−0.69^**^	–	0.55^*^
*Parietal (0–3)*	0.53^*^	−0.57^*^	–	0.55^*^
*Temporal (0–3)*	0.55^*^	−0.53^*^	–	0.64^*^
*Occipital (0–3)*	0.57^*^	−0.64^*^	–	0.39
**Layers**				
*PV (0–4)*	0.63^*^	−0.68^**^	–	0.62^*^
*M (0–4)*	0.72^**^	−0.78^***^	–	0.68^**^
*CSC (0–4)*	0.60^*^	−0.58^*^	−0.33	0.54^*^
**Subcortical structures**				
*Lenticular nc (0–1)*	–	–	−0.32	–
*Caudate nc (0–1)*	0.35	–	–	0.35
*PLIC (0–1)*	0.45	−0.57^*^	–	0.58^*^
*Thalamus (0–1)*	0.45	−0.57^*^	–	0.58^*^
*Brainstem (0–1)*	0.45	−0.57^*^	–	0.58^*^
**Corpus callosum (0–3)**	–	–	0.62^*^	–
**LESION EXTENT (i.e., global and total scores)**				
Hemispheric score (0–12)	0.68^**^	−0.72^**^	–	0.64^*^
Subcortical score (0–5)	0.39	−0.38	–	0.52^*^
Ipsilesional total (0–17)	0.61^*^	−0.64^*^	–	0.63^*^
Contralesional total (0–17)	0.47	−0.71^**^	–	0.33
Global score (0–40)	0.90^***^	−0.82^***^	–	0.59^*^

a biserial correlation coefficient; CDGM, cortical and deep gray matter; TimeVmax, timing to maximum velocity; Vmax, maximum velocity; TS, trajectory straightness; PV, periventricular; M, middle white matter; CSC, cortico-subcortical; nc, nucleus; PLIC, posterior limb of the internal capsule;

* p < 0.05;

** p < 0.01;

*** p < 0.001;

– *, no correlation (r < 0.30); 0.30–0.50, low correlation; 0.50–0.70, moderate correlation; 0.70–0.90, high correlation; moderate correlations are highlighted in bold*.

#### Movement pathology

In the **PWM group**, only a few and low correlations were found (Table 3). Damage to the PLIC was correlated with higher APS (r_b_ = 0.36) and increased movement deviations of elbow flexion/extension, shoulder elevation and trunk rotation (r_s_ = 0.35, 0.42, and 0.38, respectively). Low correlations were also found between higher subcortical scores and increased AVS of elbow flexion/extension (r_s_ = 0.40), between higher global scores and scapula medial/lateral rotation (r_s_ = 0.39) and between involvement of the thalamus and trunk rotation (r_s_ = 0.44). In contrast, damage to the brainstem was correlated with lower AVS of trunk flexion/extension (r_s_ = −0.37) and damage to the corpus callosum showed a negative correlation with AVS of scapula protraction/retraction and trunk lateral bending (r_s_ = −0.42 and r_s_ = −0.45, respectively).

**Table 3:** Correlation coefficients between the APS and AVS and brain lesion scores in the PWM group (*N* = 33).

**(A) APS and AVS of wrist and elbow**					
	**APS**^a^	**WRIST**^b^		**ELBOW**^b^	
		**Fl Ext**	**Uln Rad Dev**	**Pro Sup**	**Fl Ext**
**LESION LOCATION**					
**Lobes**					
*Frontal (0–3)*	–	–	–	–	–
*Parietal (0–3)*	–	–	–	–	–
*Temporal (0–3)*	–	–	–	–	–
*Occipital (0–3)*	–	–	–	–	–
**Layers**					
*PV (0–4)*	–	–	–	–	–
*M (0–4)*	–	–	–	–	–
*CSC (0–4)*	–	–	–	–	–
**Subcortical structures**					
*Lenticular nc (0–1)*	–	–	–	–	–
*Caudate nc (0–1)*	–	–	–	–	–
*PLIC (0–1)*	0.36^*^	–	0.31	–	0.35^*^
*Thalamus (0–1)*	–	–	–	–	–
*Brainstem (0–1)*	–	–	–	–	–
**Corpus Callosum (0**–**3)**	–	–	–	–	–
**LESION EXTENT (i.e., global and total scores)**					
HS (0–12)	–	–	–	–	–
SS (0–5)	–	–	–	–	0.40^*^
Ipsi total (0–17)	–	–	–	–	–
Contra total (0–17)	–	–	–	–	–
Global score (0–40)	–	–	–	–	–

**Table:** 

**(B) AVS of shoulder, scapula and trunk**									
	**SHOULDER**^c^			**SCAPULA**^c^			**TRUNK**^c^		
	**Elev Plane**	**Elev**	**Rot**	**Pro/Retr**	**Med/Lat Rot**	**Tilt**	**Fl Ext**	**Lat Fl**	**Rot**
**LESION LOCATION**									
**Lobes**									
*Frontal (0–3)*	–	–	–	–	–	–	–	–	–
*Parietal (0–3)*	–	–	–	–	–	0.33	–	–	–
*Temporal (0–3)*	–	–	–	–	–	–	–	–	–
*Occipital (0–3)*	–	–	–	–	–	–	–	–	0.30
**Layers**									
*PV (0–4)*	–	–	–	–	–	–	–	–	0.32
*M (0–4)*	–	–	–	–	–	–	–	–	–
*CSC (0–4)*	–	**–**	–	–	–	–	–	–	–
**Subcortical structures**									
*Lenticular nc (0–1)*	–	–	–	–	–	–	–	–	–
*Caudate nc (0–1)*	–	–	–	–	–	–	–	–	–
*PLIC (0–1)*	–	0.42^*^	–	–	–	–	–	–	0.38^*^
*Thalamus (0–1)*	–	–	–	–	–	–	–	–	0.44^*^
*Brainstem (0–1)*	–	0.32	–	–	–	–	−0.37^*^	–	–
**CC (0–3)**	–	–	–	−0.42^*^	0.32	–	–	−0.45^*^	–
**LESION EXTENT (i.e., GLOBAL AND TOTAL SCORES)**									
HS (0–12)	–	–	–	–	–	–	–	–	–
SS (0–5)	–	–	–	–	–	–	–	–	–
Ipsi total (0–17)	–	–	–	–	–	–	–	–	–
Contra total (0–17)	–	–	–	–	–	–	–	–	–
Global score (0–40)	–	–	–	–	0.39^*^	–	–	–	–

a biserial correlation coefficient;

b spearman correlation coefficient; APS, arm profile score; AVS, arm variable scores; Fl, flexion; Ext, extension; Uln, ulnar; Rad, radial; Dev, deviation; Pro, pronation; Sup, supination; PV, periventricular; M, middle white matter; CSC, cortico-subcortical; nc, nucleus; PLIC, posterior limb of the internal capsule; HS, hemispheric score; SS, subcortical score; ipsi, ipsilesional; contra, contralesional;

* p < 0.05;

– *, no correlation (r < 0.30); 0.30–0.50, low correlation*.

c spearman correlation coefficient; AVS, arm variable scores; Elev, elevation; Rot, rotation; Pro, protraction; Retr, retraction; Med, medial; Lat, lateral; Tilt, tilting; Fl, flexion; Ext, extension; PV, periventricular; M, middle white matter; CSC, cortico-subcortical; nc, nucleus; PLIC, posterior limb of the internal capsule; CC, corpus callosum; HS, hemispheric score; SS, subcortical score; ipsi, ipsilesional; contra, contralesional;

* p < 0.05;

– *, no correlation (r < 0.30); 0.30–0.50, low correlation*.

In children with **CDGM lesions**, more and stronger correlations were found between the summary indices and brain lesion location and extent (Table 4). These correlations were most pronounced for the APS (r_b_ = 0.36–0.63) and the AVS of the wrist and elbow (r_s_ = 0.34–0.65). Proximally, correlations were rather scarce and mostly low. Regarding *lesion location*, damage to the temporal lobe and involvement of the cortico-subcortical layer correlated moderately with increased APS and AVS of wrist flexion/extension, elbow pronation/supination and elbow flexion/extension (r_s_ = 0.50–0.65). Further, positive, moderate correlations were found between involvement of the PLIC, thalamus and brainstem and higher AVS of wrist and elbow flexion/extension (r_s_ = 0.50–0.59). Proximally, positive moderate correlations were seen between damage to the corpus callosum and higher AVS of scapular protraction/retraction (r_s_ = 0.53) and between damage to the caudate nucleus and the AVS of trunk flexion/extension (r_s_ = 0.59). Regarding *lesion extent*, moderate correlations were found between higher ipsilesional hemispheric scores and increased AVS of elbow pronation/supination (r_s_ = 0.54). Higher ipsilesional subcortical scores were further moderately correlated with increased APS and AVS of wrist and elbow flexion/extension (r_s_ = 0.51–0.62). Higher ipsilesional total scores were moderately correlated with increased APS and AVS of elbow pronation/supination (r_b_ = 0.51 and r_s_ = 0.50, respectively). Finally, also two negative moderate correlations were found, i.e., between damage to the lenticular nucleus and the AVS of scapular protraction/retraction (r_s_ = −0.58) and between damage to the caudate nucleus and the AVS of shoulder elevation plane (r_s_ = −0.59).

**Table 4:** Correlation coefficients between the APS and AVS and brain lesion scores in the CDGM group (*N* = 15).

**(A) APS and AVS of wrist and elbow**					
	**APS^a^**	**WRIST^b^**		**ELBOW^b^**	
		**Fl Ext**	**Uln Rad Dev**	**Pro Sup**	**Fl Ext**
**LESION LOCATION**					
**Lobes**					
*Frontal (0–3)*	0.36	–	–	**0.51**	0.35
*Parietal (0–3)*	–	–	–	–	–
*Temporal (0–3)*	0.63^*^	0.64^**^	–	0.65^**^	0.55^*^
*Occipital (0–3)*	–	–	–	0.41	–
**Layers**					
*PV (0–4)*	0.43	0.39	–	0.48	–
*M (0–4)*	0.41	–	–	0.49	–
*CSC (0–4)*	0.53^*^	**0.50**	–	0.58^*^	**0.50**
**Subcortical structures**					
*Lenticular nc (0–1)*	–	0.42	–	–	–
*Caudate nc (0–1)*	**0.50**	0.43	0.52^*^	0.39	0.40
*PLIC (0–1)*	0.46	**0.50**	–	0.41	0.59^*^
*Thalamus (0–1)*	0.46	**0.50**	–	0.41	0.59^*^
*Brainstem (0–1)*	0.46	**0.50**	–	0.41	0.59^*^
**Corpus Callosum (0–3)**	–	–	–	–	–
**LESION EXTENT (i.e., GLOBAL AND TOTAL SCORES)**					
HS (0–12)	0.47	0.41	–	0.54^*^	0.34
SS (0–5)	0.52^*^	0.62^*^	–	0.37	**0.51**
Ipsi total (0–17)	**0.51**	0.45	–	**0.50**	0.34
Contra total (0–17)	–	–	–	–	–
Global score (0–40)	–	–	–	0.35	–

**Table:** 

**(B) AVS of shoulder, scapula and trunk**									
	**SHOULDER**^c^			**SCAPULA**^c^			**TRUNK**^c^		
	**Elev Plane**	**Elev**	**Rot**	**Pro/Retr**	**Med/Lat Rot**	**Tilt**	**Fl Ext**	**Lat Fl**	**Rot**
**LESION LOCATION**									
**Lobes**									
*Frontal (0–3)*	–	–	–	–	–	–	–	–	–
*Parietal (0–3)*	–	–	–	0.33	–	–	–	0.32	–
*Temporal (0–3)*	–	0.36	–	–	–	–	–	–	–
*Occipital (0–3)*	–	0.38	–	–	–	–	–	–	–
**Layers**									
*PV (0–4)*	–	–	–	–	–	–	–	–	–
*M (0–4)*	–	–	–	0.38	–	–	–	–	–
*CSC (0–4)*	–	–	–	–	–	–	–	–	–
**Subcortical structures**									
*Lenticular nc (0–1)*	–	–	–	–	−0.58^*^	–	–	–	–
*Caudate nc (0–1)*	–	−0.59^*^	–	0.39	–	–	–	0.59^*^	–
*PLIC (0–1)*	–	–	–	–	–	0.36	–	–	–
*Thalamus (0–1)*	–	–	–	–	–	0.36	–	–	–
*Brainstem (0–1)*	–	–	0.32	–	–	0.36	–	–	–
**CC (0–3)**	–	–	–	0.53^*^	0.47	0.34	–	–	–
**LESION EXTENT (i.e., global and total scores)**									
HS (0–12)	–	–	–	–	–	–	–	–	–
SS (0–5)	–	–	–	–	–	–	0.31	–	–
Ipsi total (0–17)	–	–	–	–	–	–	–	–	–
Contra total (0–17)	–	0.46	–	–	–	–	–	–	–
Global score (0–40)	–	–	–	0.36	–	–	0.32	–	–

a biserial correlation coefficient;

b spearman correlation coefficient; APS, arm profile score; AVS, arm variable scores; Fl, flexion; Ext, extension; Uln, ulnar; Rad, radial; Dev, deviation; Pro, pronation; Sup, supination; PV, periventricular; M, middle white matter; CSC, cortico-subcortical; nc, nucleus; PLIC, posterior limb of the internal capsule; HS, hemispheric score; SS, subcortical score; ipsi, ipsilesional; contra, contralesional;

* p < 0.05;

– *, no correlation (r < 0.30); 0.30–0.50, low correlation; 0.50–0.70, moderate correlation; moderate correlations are highlighted in bold*.

c spearman correlation coefficient; AVS, arm variable scores; Elev, elevation; Rot, rotation; Pro, protraction; Retr, retraction; Med, medial; Lat, lateral; Tilt, tilting; Fl, flexion; Ext, extension; PV, periventricular; M, middle white matter; CSC, cortico-subcortical; nc, nucleus; PLIC, posterior limb of the internal capsule; CC, corpus callosum; HS, hemispheric score; SS, subcortical score; ipsi, ipsilesional; contra, contralesional;

* p < 0.05;

– *, no correlation (r < 0.30); 0.30–0.50, low correlation; 0.50–0.70, moderate correlation; moderate correlations are highlighted in bold*.

### Multiple regression

The LASSO regression revealed the ipsilesional temporal lobar score with lesion timing as interactor and the PLIC as the strongest predictors of the APS. Together, these variables explained 34% of the variance in APS, with damage to the temporal lobe combined with lesion timing as the strongest contributor (27%, *p* = 0.002; PLIC 7%, *p* = 0.04).

## Discussion

This study was the first to investigate differences in UL kinematics, quantified by means of an UL 3DMA between children with PWM and CDGM lesions and to explore the impact of lesion location and extent on UL kinematics within each lesion timing group. These insights are critical to identify the underlying neural mechanisms of UL movement pathology. We found significant differences in UL kinematics between both groups, whereby children with CDGM lesions exhibit more movement pathology, and further showed that lesion location and extent were more strongly related with UL kinematics in the CDGM group, except for proximal movement pathology. The PLIC was identified as the only important brain structure for UL movement pathology irrespective of lesion timing.

The results of this study demonstrated for the first time that lesion timing influences UL movement pathology in children with uCP. Children with CDGM lesions moved slower, reached their maximum velocity earlier and moved less smooth compared to children with PWM lesions. Additionally, the A-MAP showed that children with CDGM lesions have more deviant movement patterns than children with PWM lesions. Differences were significant for total movement pathology (APS) and movement deviations of wrist flexion/extension and shoulder elevation as well as a borderline significant difference for elbow pronation/supination. Inspection of the waveforms showed that the CDGM group executed the RGV task with more wrist flexion and elbow pronation and reached their maximal shoulder elevation earlier compared to the PWM group (Supplementary Material SF1). Current findings confirmed our hypothesis that UL kinematics are more deviant in children with later lesions. These findings are in line with previous studies reporting that children with CDGM lesions have a more impaired UL function, assessed with clinical outcomes, compared to children with PWM lesions (Feys et al., 2010; Holmström et al., 2010; Holmefur et al., 2013; Mackey et al., 2014).

Secondly, correlation analysis between UL kinematics and lesion location and extent clearly showed different results for each lesion timing group. In the CDGM group, moderate to high correlations were found between increasing brain damage and more deviating spatiotemporal parameters, while no correlations were found in the PWM group. Previously, only Van Der Heide et al. (2005) investigated the relation between brain lesion severity and trajectory straightness in 50 children with uCP during a reach-to-grasp task and reported only low correlations. However, these authors did not make a distinction depending on lesion timing, which might explain why they could not demonstrate a clear relationship. In addition, brain lesion severity was scored on a 3-point scale based on ultrasound images only, which is known to be less accurate to detect diffuse white matter injury compared to MRI (Inder et al., 2003).

Furthermore, more and stronger correlations between lesion location and extent and UL movement pathology were found in children with CDGM lesions compared to children with PWM lesions, however, only for the distal joints. In the CDGM group, involvement of the temporal lobe, cortico-subcortical layer, PLIC, thalamus, brainstem as well as higher ipsilesional hemispheric, ipsilesional subcortical, and ipsilesional total scores were correlated with increased movement pathology of wrist flexion/extension, elbow pronation/supination and elbow flexion/extension. Interestingly, despite moderate correlations between lesion location and extent and distal UL movement pathology, correlations between lesion location and extent and shoulder, scapula and trunk kinematics were scattered and low. Klingels et al. (2012) have previously reported that motor impairments, such as increased muscle tone and weakness were most prominent at the wrist and elbow, compared to the shoulder muscles. Hence, proximal UL movement pathology may not solely be affected by the impairments in those regions, but also by the posture of the child and the compensation strategies used to overcome the more pronounced distal deficits (Jaspers et al., 2011a; Simon-Martinez et al., 2017). This might also explain the few unexpected correlations implying a relation between increased brain damage and less proximal UL movement pathology. Overall, these findings suggest that structural brain damage impact less on shoulder, scapula and trunk kinematics compared to the wrist and elbow in children with CDGM lesions. In contrast, in the PWM group only a few and low correlations were found, mostly between damage to the PLIC and UL movement pathology. These findings correspond to a recent study of our research group, whereby lesion location and extent were more strongly related with clinical measures of UL function in children with CDGM lesions than children with PWM lesions (Mailleux et al., 2017b). There was an overlap of only five participant between both studies. It was hypothesized that in children with PWM lesions, other brain lesion characteristics, such as white matter microstructure and type of corticospinal tract (re)organization, might need to be considered next to lesion location and extent when investigating structure-function relationships in this group. Secondly, white matter damage in children with PWM lesions might allow a higher gray matter plasticity compared to children with CDGM lesions, which could also explain the lower correlations in the PWM group.

Diffusion-weighted MRI is found to be more sensitive in detecting subtle white matter abnormalities compared to a conventional MRI scan (Son et al., 2007) and is particularly well-suited to visualize white matter microstructure. Previous studies have already shown that white matter microstructural properties of both the corticospinal tract and thalamocortical tracts are associated with UL function in children with uCP (Holmström et al., 2011; Rose et al., 2011; Mackey et al., 2014; Tsao et al., 2014, 2015). Additionally, the type of corticospinal tract (re)organization (i.e., contralateral, ipsilateral or bilateral) has also been shown to play a role in defining UL function (Staudt et al., 2004; Holmström et al., 2010; Jaspers et al., 2016), which can be assessed using transcranial magnetic stimulation. Overall, children with an ipsilateral tract reorganization have a more impaired UL function compared to contralateral or bilateral tract reorganization. However, the efficacy of corticospinal tract reorganization largely depends on the timing of the lesion (Staudt et al., 2004). Due to the earlier onset of PWM lesions, these children have a higher efficacy of reorganizing to the contralesional hemisphere. Hence, future multimodal imaging studies might aid in unraveling the multifactorial interaction of these brain lesion characteristics on UL movement pathology in children with uCP, particularly in children with PWM lesions.

In the total group, regression analysis confirmed the importance of the PLIC for total UL movement pathology irrespective of lesion timing. Correspondingly, in both groups, damage to the PLIC was related with UL movement pathology. This is not surprising since the main motor pathways for voluntary motor control, i.e., the corticospinal tract, pass through the PLIC in order to continue its path down to the spinal cord. Together, these results point to the importance of this brain structure for UL motor function, which is in line with previous work (Holmström et al., 2011; Mackey et al., 2014; Dinomais et al., 2015; Mailleux et al., 2017b). Nevertheless, regression analysis identified the ipsilesional temporal lobe as the strongest predictor to explain the variance in APS, but only when lesion timing was taken into account. Correlation analysis showed that damage to the temporal lobe was moderately related with the APS only in the CDGM group. Accordingly, two other studies also retained the temporal lobe as a significant contributor for UL function in children with CDGM lesions (Pagnozzi et al., 2016; Mailleux et al., 2017b). The temporal lobe is associated with action recognition, in case the action is related to the manipulation of a certain tool (Quandt and Chatterjee, 2015). Also, visual stimuli that confer information about the external world are processed in the temporal lobe (Quandt and Chatterjee, 2015). Such stimuli are needed in order to properly execute the task RGV. This task requires information about the distance to the object and the circumference of the object to be grasped in terms of anticipatory hand shaping. Hence, these functions of the temporal lobe might thus explain why this region is important for UL motor function.

In total, regression analysis revealed a substantial 34% of the variance in APS. Still, clearly other factors will play an additional role in determining UL movement pathology. For example, different neural mechanisms defining UL movement pathology or the influence of compensation strategies at the proximal joints on total movement pathology as mentioned previously. For future studies, it might be interesting to develop a distal and proximal APS to allow for a better distinction between distal and proximal UL movement pathology. Hence, we would expect that for the distal APS a larger part of the variance could be explained by structural brain damage compared to the proximal APS. Furthermore, this 3DMA protocol does not capture fine finger and thumb movements, which are indispensable for UL function. Taking into account these movements might allow a more in-depth analysis of the relation between structural brain damage and UL movement pathology.

Finally, we also explored the relation between contralesional brain damage and UL kinematics. Despite a diagnosis of uCP, bilateral lesions in these children are not a rare phenomenon. Previous studies reported frequencies of up to 50% (Feys et al., 2010; Holmström et al., 2010; Holmefur et al., 2013). In this study, bilateral lesions were seen in 33% of the children with PWM lesions (*N* = 11) as well as with CDGM lesions (*N* = 5). For the spatiotemporal parameters, one high correlation was found between contralesional brain damage and timing of maximum velocity in the CDGM group. However, no further correlations were found between contralesional brain damage (i.e., contralesional total score) and the summary indices, except for one low correlation with the AVS of shoulder elevation in the CDGM group. Hence, this might imply that contralesional brain damage does not affect movement pathology of the impaired UL. Correspondingly, previous studies have also shown no differences in bimanual performance between children with unilateral and bilateral lesions (Holmefur et al., 2013; Mailleux et al., 2017b).

Nevertheless, this study also has a few limitations to address. First, children with botulinum toxin-A injections were included in case these injections occurred at least 6 months prior to testing. However, even though the effect of botulinum injections is temporary, nothing is currently known on whether (repeated) botulinum toxin injections could permanently change UL movement patterns. Secondly, we decided to include only children with PWM and CDGM lesions as these types of lesions occur most often (50% and 20–30% respectively, Krägeloh-Mann and Horber, 2007; Feys et al., 2010). Consequently, current study results cannot be generalized to children with brain maldevelopments, which occur in 10–15% of the cases. We further must acknowledge that a correction for multiple testing was not applied, increasing the chance of a type 1 error. However, we decided not to apply a correction due to the explorative nature of the study and considered it clinically more meaningful to interpret correlations according to their strength. Although this study was applied on a large group of children with uCP (*N* = 48), classifying them according to lesion timing retained only 15 children in the CDGM group. Nevertheless, this study was the first to explore the relation between lesion location and extent and UL kinematics taking into account lesion timing, suggesting that the underlying neural mechanisms of UL movement pathology differ between children with PWM and CDGM lesions. Moreover, the current study findings emphasize the importance of taking into account brain lesion characteristics when interpreting the clinical picture of UL function in children with uCP, particularly in children with CDGM lesions and temporal lobe involvement. The higher amount of movement pathology in children with CDGM lesions compared to PWM lesions may be caused by the more severe underlying sensorimotor impairments in the CDGM group (Mailleux et al., 2017a; Simon-Martinez et al., 2017), which was also confirmed in our study (see Supplementary Material ST1). Thus, these children are more at risk of developing secondary musculoskeletal problems. In these children, therapy could focus on improving active range of motion and reducing muscle tone (e.g., botulinum toxin injections) in order to improve UL movement patterns. Hence, the findings of this study may aid in guiding patient selection for interventions and optimizing individualized intervention strategies. In addition, there is a need to further explore and validate the utility of brain lesion characterization using early imaging to predict motor outcome at an older age and thus, improve the prognostic information for patients and clinicians.

## Conclusion

This study demonstrated that children with CDGM lesions have more deviant UL kinematics compared to children with PWM lesions. Secondly, more prominent relationships were shown between lesion location and extent and UL kinematics in the CDGM group compared to the PWM group. This finding might imply that the underlying neural mechanisms of UL movement pathology differ between children with different lesion timings. However, multimodal imaging studies are needed to clarify whether white matter microstructure and type of corticospinal tract (re)organization can explain more of the variance in APS. The PLIC was the only significant predictor for UL movement pathology irrespective of lesion timing. Finally, it seemed that proximal UL movement pathology is less influenced by lesion location and extent compared to movement deviations at the wrist and elbow, particularly in children with CDGM lesions. Other factors, such as posture and compensation strategies might play a larger role in defining proximal UL movement pathology. Although this needs further investigation, the finding that structural brain damage mostly relates to distal UL movement pathology compared to proximal UL kinematics, clearly demonstrated the added value of using 3DMA to investigate structure-function relationships.

## Author contributions

This study was designed by LM, KK, EO, and HF. LM, CS-M, and EJ were responsible for all data collection and analysis. EO scored all scans with the sqMRI scale. All authors contributed to the interpretation of the results and gave their critical views regarding the revision and editing of the manuscript written by LM. All authors approved the final version of the manuscript and agreed to be accountable for the content of the study.

### Conflict of interest statement

The authors declare that the research was conducted in the absence of any commercial or financial relationships that could be construed as a potential conflict of interest.

## Footnotes

^1^https://github.com/u0078867/ulema-ul-analyzer
