# Supplementary material for: Structural Brain Damage and Upper Limb Kinematics in Children with Unilateral Cerebral Palsy
Source: Front Hum Neurosci. 2017 Dec 12;11:607. doi: 10.3389/fnhum.2017.00607 (PMC5733007; doi:10.3389/fnhum.2017.00607)
Supplement: Supplementary file 1 [file Table1.docx]

Supplementary Material

Structural brain damage and upper limb kinematics in children with unilateral cerebral palsy.

**Mailleux Lisa^*^, Simon-Martinez Cristina, Klingels Katrijn, Jaspers Ellen, Desloovere Kaat, Demaerel Philippe, Fiori Simona, Guzzetta Andrea, Ortibus Els, Feys Hilde.**

*** Correspondence:** lisa.mailleux@kuleuven.be

## 1. Supplementary Tables

| **ST 1. Descriptive statistics of clinical outcome measures and statistical comparison between the PWM group (N=33) and CDGM group (N=15).** | | | | |
| --- | --- | --- | --- | --- |
|  |  | **PWM group** | **CDGM group** | **p-values** |
| Muscle Tone^a^ | Me (P25-P75) | 7 (5-8.5) | 8.25 (6.5-10.5) | 0.11 |
| Muscle Strength^a^ | Me (P25-P75) | 34 (32-35.5) | 31.75 (30-33) | **0.01** |
| Grip Strength (%)^a^ | Me (P25-P75) | 45.45 (30.19-69.35) | 25.86 (17.07-32.5) | **0.002** |
|  |  |  |  |  |
| 2PD^b^ |  |  |  | **<0.0001** |
| Absent | N(%) | 1 (3%) | 8 (57%) |  |
| Impaired | N(%) | 4 (12%) | 3 (21%) |  |
| Normal | N(%) | 28 (85%) | 3 (21%) |  |
| Stereognosis^b^ |  |  |  | **<0.0001** |
| Absent | N(%) | 2 (6%) | 10 (67%) |  |
| Impaired | N(%) | 8 (24%) | 3 (20%) |  |
| Normal | N(%) | 23 (70%) | 2 (13%) |  |
|  |  |  |  |  |
| AHA^a^ | Me (P25-P75) | 65 (59-77) | 54.5 (49-55) | **0.0008** |
| MA2 ROM^a^ | Me (P25-P75) | 81.48 (62.96-85.19) | 50.93 (40.47-58.33) | **0.0002** |
| MA2 ACC^a^ | Me (P25-P75) | 100 (92-100) | 84 (73.91-88) | **<0.0001** |
| MA2 DEX^a^ | Me (P25-P75) | 75 (68.75-87.5) | 48.08 (37.5-56.26) | **0.0007** |
| MA2 FL^a^ | Me (P25-P75) | 76.19 (66.67-95.24) | 64.29 (57.14-76.19) | **0.02** |
| Grip strength is displayed as the ratio of the impaired versus the less impaired hand; 2PD, two-point discrimination; AHA, Assisting Hand Assessment, MA2, Melbourne Assessment 2; ROM, range of motion; ACC, accuracy; DEX, dexterity; FL, fluency; N, number; Me, median; P, percentile; PWM, periventricular white matter; CDGM, cortical and deep grey matter; ^a^, Wilcoxon rank sum test; ^b^, Fisher’s exact test; bold, p<0.05. | | | | |
