# Supplementary material for: Structural Brain Damage and Upper Limb Kinematics in Children with Unilateral Cerebral Palsy
Source: Front Hum Neurosci. 2017 Dec 12;11:607. doi: 10.3389/fnhum.2017.00607 (PMC5733007; doi:10.3389/fnhum.2017.00607)
Supplement: Supplementary file 2 [file Table2.DOCX]

Supplementary Material

Structural brain damage and upper limb kinematics in children with unilateral cerebral palsy.

**Mailleux Lisa^*^, Simon-Martinez Cristina, Klingels Katrijn, Jaspers Ellen, Desloovere Kaat, Demaerel Philippe, Fiori Simona, Guzzetta Andrea, Ortibus Els, Feys Hilde.**

*** Correspondence:** lisa.mailleux@kuleuven.be

## 1. Supplementary Tables

| **ST2. Statistical comparison of the APS (X, SD) and AVS (Me, IQR) between the PWM (N=33) and the CDGM (N=15) group.** | | | | |
| --- | --- | --- | --- | --- |
|  |  | PWM group | CDGM group | p-value |
| APS |  | **16.50 (5.95)** | **22.20 (5.63)** | **0.003** |
| WRIST | flexion/extension | **15.52 (10.65 - 28.40)** | **33.62 (19.93 - 33.62)** | **0.01** |
|  | ulnar/radial deviation | 8.38 (6.45 - 19.23) | 10.17 (5.41 - 25.69) | 0.71 |
| ELBOW | pronation/supination | 22.38 (13.24 - 36.08) | 40.32 (22.20 - 56.04) | 0.08 |
|  | flexion/extension | 21.10 (18.30 - 27.87) | 24.03 (17.33 - 36.74) | 0.44 |
| SHOULDER | elevation plane | 12.53 (8.06 - 15.37) | 17.48 (11.82 - 23.81) | 0.09 |
|  | elevation | **11.50 (8.52 - 14.37)** | **15.34 (11.18 - 16.27)** | **0.05** |
|  | rotation | 14.04 (10.50 - 23.19) | 20.69 (11.13 - 29.68) | 0.22 |
| SCAPULA | protraction/retraction | 5.95 (3.91 - 8.81) | 7.53 (5.32 - 10.98) | 0.12 |
|  | medial/lateral rotation | 9.21 (4.32 - 15.15) | 8.48 (6.66 - 17.53) | 0.30 |
|  | anterior/posterior tilting | 5.59 (4.46 - 9.91) | 7.56 (3.53 - 13.34) | 0.34 |
| TRUNK | flexion/extension | 4.81 (2.81 - 6.59) | 5.57 (2.97 - 8.52) | 0.37 |
|  | lateral bending | 4.28 (2.08 - 6.09) | 5.25 (3.58 - 7.43) | 0.15 |
|  | rotation | 5.47 (4.35 - 7.78) | 8.57 (4.36 - 13.24) | 0.15 |
| APS, Arm Profile Score; AVS, Arm Variable Score; X, mean; SD, standard deviation; Me, median; IQR, interquartile range; PWM, periventricular white matter; CDGM cortical and deep gray matter. | | | | |
