# Supplementary figures and images for: Structural Brain Damage and Upper Limb Kinematics in Children with Unilateral Cerebral Palsy
Source: Front Hum Neurosci. 2017 Dec 12;11:607. doi: 10.3389/fnhum.2017.00607 (PMC5733007; doi:10.3389/fnhum.2017.00607)

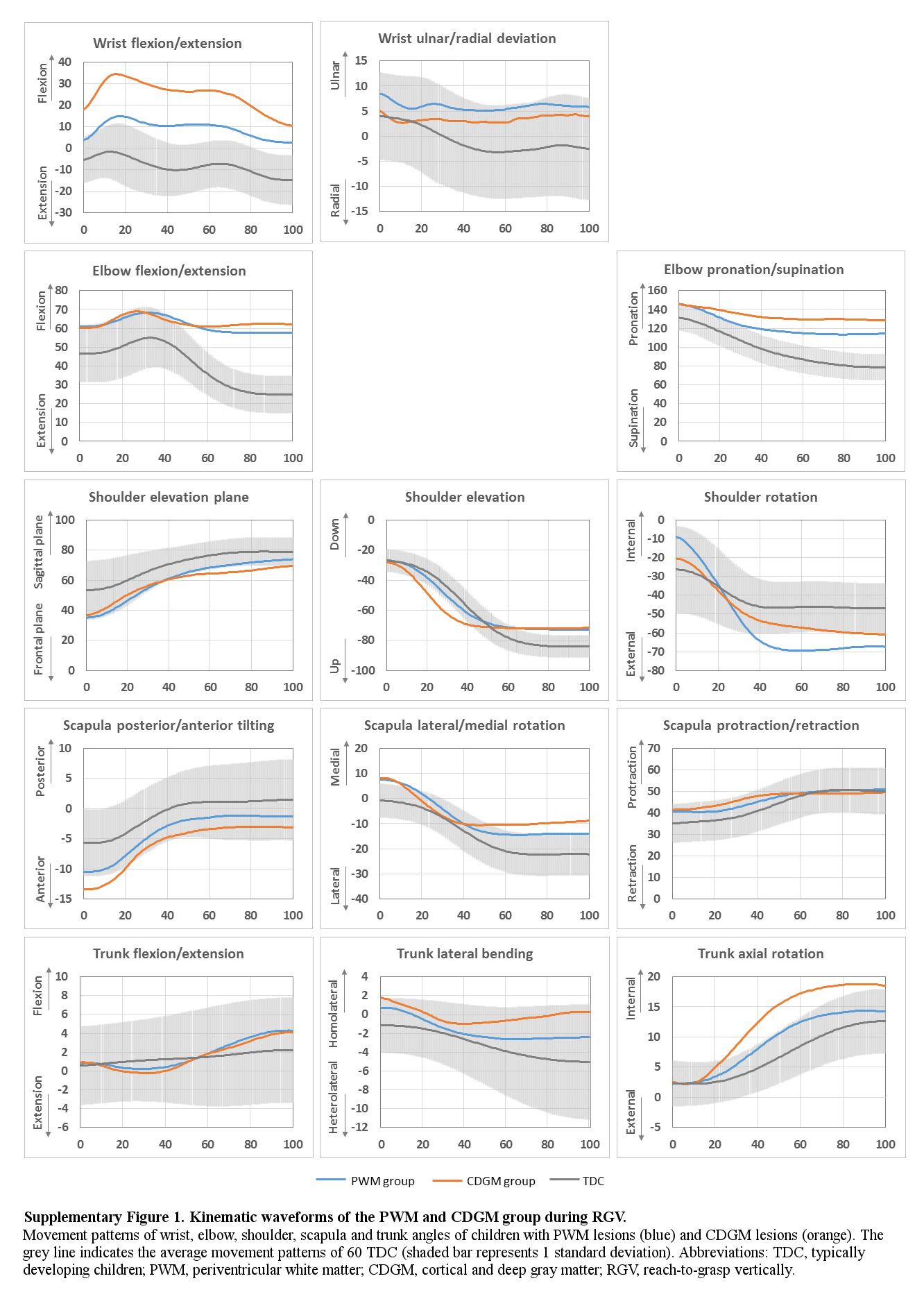

Supplement: Supplementary file 3 [file Image1.TIFF]
